# Porous Silica Microspheres with Immobilized Titania Nanoparticles for In‐Flow Solar‐Driven Purification of Wastewater

**DOI:** 10.1002/gch2.202000116

**Published:** 2021-01-27

**Authors:** Ana C. Marques, Mário Vale, Daniel Vicente, Murielle Schreck, Elena Tervoort, Markus Niederberger

**Affiliations:** ^1^ CERENA DEQ Instituto Superior Técnico Universidade de Lisboa Avenida Rovisco Pais Lisboa 1049‐001 Portugal; ^2^ Laboratory for Multifunctional Materials Department of Materials ETH Zürich, Vladimir‐Prelog‐Weg 5 Zürich 8093 Switzerland

**Keywords:** microspheres, photocatalysis, porous silica, solar light, sol–gel, titania nanoparticles

## Abstract

In this paper, inorganic silica microspheres with interconnected macroporosity are tested as a platform for designing robust and efficient photocatalytic systems for a continuous flow reactor, enabling a low cost and straightforward purification of wastewater through solar‐driven photocatalysis. The photocatalytically active microspheres are prepared by wet impregnation of porous silica scaffolds with Trizma‐functionalized anatase titania (TiO_2_) nanoparticles (NPs). NPs loading of 22 wt% is obtained in the form of a thin and well‐attached layer, covering the external surface of the microspheres as well as the internal surface of the pores. The TiO_2_ loading leads to an increase of the specific surface area by 26%, without impacting the typically interconnected macroporosity (**≈**60%) of the microspheres, which is essential for an efficient flow of the pollutant solution during the photocatalytic tests. These are carried out in a liquid medium for the decomposition of methyl orange and paracetamol. In addition to photocatalytic activity under continuous flow, the microspheres offer the advantage that they can be easily removed from the reaction medium, which is an appealing aspect for industrial applications. In this work, the typical issues of TiO_2_ NPs photocatalysts are circumvented, without the need for elaborate chemistries, and for low availability and expensive raw materials.

## Graphical abstract

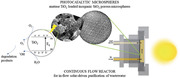

## 1 Introduction

Removing organic pollutants from wastewater is a critical need and a challenge as well. A first option is their adsorption on the surface of an active element, such as activated carbon or bentonite,^[^ ^1^, ^2^ ^]^ or a technique based on reverse osmosis.^[^ ^3^, ^4^ ^]^ However, they are intrinsically discontinuous methods. The adsorbent or membrane must be reactivated or regenerated regularly in a more frequent fashion for high pollutant's concentrations. Moreover, none of these methods involve degradation of the contaminants as it happens for anaerobic biological treatments.^[^ ^5^ ^]^ Techniques based on electrochemical processes or ozonation include large energy consumption and high operating cost, which significantly limits their applications.

Heterogeneous photocatalysis, an advanced oxidation process, appears as a good alternative for the treatment of organic pollutants due to its green feature, the economic and efficient capability of degrading a wide range of water‐dispersed contaminants into readily biodegradable and less toxic compounds.

TiO_2_ is one of the most commonly used photocatalysts due to its advantages of high stability, non‐toxicity, low cost, and well‐controlled, straightforward synthesis procedure. However, it displays a relatively large bandgap, which makes it active under ultraviolet (UV) light mostly. To enable the use of visible (vis) light‐driven photocatalysis, TiO_2_ has been doped with metals, such as noble and transition metals, or non‐metals (N, B, S, F, and C). The noble metals (such as Ag, Au, Pd, and Pt) present some advantages such as the possibility to absorb the vis light due to the surface plasmon resonance.^[^ ^6^, ^7^ ^]^ Still, the high cost associated with these materials should be considered a disadvantage, unless they are used at a minimal concentration. The transition metals, on the other hand, might tend to leach, leading to the fast deactivation of the catalyst and constituting a second source of pollution, requiring the removal of dissolved metals from treated water.^[^ ^8^ ^]^ Non‐metals, such as N, lead to the narrowing of TiO_2_ bandgap, which limits the recombination phenomenon.^[^ ^9^ ^]^ Still, in some cases, their preparation method involves high‐energy costs and requires specialized equipment.

Therefore, there is a high interest for achieving photocatalytic degradation under vis light irradiation, when an intrinsic (non‐doped) TiO_2_ photocatalyst is used.

The upscaling and modification of a non‐aqueous sol–gel based synthesis route to produce surface‐functionalized TiO_2_ (anatase) NPs, developed by Niederberger et al.,^[^ ^10^ ^]^ is employed in this work. The as‐synthesized NPs exhibit the advantage of being already crystalline (anatase), without the use of heat treatment, and controlled crystallite size (only a few nm size) and surface composition, displaying photocatalytic performance.^[^ ^11^ ^]^ To further increase their photocatalytic efficiency, they have been assembled into an aerogel,^[^ ^12^ ^]^ which allows them to be connected without agglomeration, but still with a high surface area and high accessibility of the active surfaces. Moreover, advanced aerogels have been developed, by co‐assembling spherical Au, Pd, and PdAu NPs with those TiO_2_ NPs and applying them for gas‐phase H_2_ generation.^[^ ^13^ ^]^ The porous structure of these aerogels promotes the light‐harvesting, reagent transport, and electron migration process, generating 3.5‐fold more hydrogen in comparison to the corresponding powders. However, aerogels tend to disintegrate in a liquid medium, being inefficient for wastewater purification (photocatalysis in liquid‐phase).

In summary, the development of efficient and robust photocatalysts that have not only suitable light harvesting property, especially in the vis region of the spectrum, but also excellent catalytic performance in liquid‐phase is still a challenge.

The use of metal oxide NPs for heterogeneous photocatalytic decomposition of various pollutants in liquid‐phase has been employed and successfully demonstrated, but has some drawbacks because the NPs are dispersed in the liquid media that they treat: a) contamination with NPs and need for sophisticated removal strategies; b) no possibility for continuous flow‐through operation, so that only small volumes of wastewater can be treated in a discontinuous (batch) way; and c) tendency to rapidly aggregate in a suspension, due to their relatively small size, considerably decreasing their effective surface area and catalytic efficiency. These disadvantages pull this technology away from practical application. Moreover, most of the works in the literature use UV irradiation, which is much more expensive and less appealing from an industrial point of view.

The above‐stated issues are potentially solved by immobilizing the photocatalytic NPs within selected or designed support materials, or scaffolds.

A new class of support material, that is, a silica‐based microscaffold of spherical shape (MSs) with custom‐tailored porosity, has been recently developed by a novel method that combines emulsion templating with an adapted sol–gel technique.^[^ ^14^, ^15^ ^]^ Its interconnected macroporosity is due to phase separation by polymerization‐induced spinodal decomposition, and a benefit of this method is that no specific gelation additives are needed to promote such phase separation.

The chemical inertness, transparency in the UV, mechanical robustness, associated with their macro and mesoporosity, make these MSs an emerging platform for achieving new and efficient engineered photocatalytic systems, by acting as a support and synergic structure where photocatalytic NPs are immobilized. While macropores act as a mass transport system for liquids and gases, increasing the accessibility of the smaller pores, mesopores are responsible for the high surface area of these materials, as well as for size and shape selectivity. So bi‐modal pore structures can significantly improve active site accessibility. The preparation of spherical‐like particles with such kind of porosity makes the straightforward assembly into infinitely many different, customized shapes (e.g., in tubular, or serpentine reactors, columns, plates, etc.) possible. Containers of any shape can be filled with spherical microscaffolds, which bring more flexibility to the process, widen the possibilities for new photocatalytic set‐ups, and enable the application of such a water purifying technology, based on photocatalysis in real‐life conditions.

After an exhaustive study of the literature toward solving the above‐stated issues, we came across a few publications regarding support materials for photocatalytic NPs, such as pyrex spheres,^[^ ^16^ ^]^ glass beads,^[^ ^17^ ^]^ glass fiber mat,^[^ ^18^ ^]^ plastic beads, or spheres,^[^ ^19^, ^20^, ^21^ ^]^ polyethylene terephthalate sheets,^[^ ^22^ ^]^ activated carbon,^[^ ^23^ ^]^ mesoporous silica SBA‐15^[^ ^24^ ^]^ and cellulose nanofibers or membranes.^[^ ^25^, ^26^, ^27^, ^28^ ^]^ This latter type of support was tested using a continuous flow‐through apparatus^[^ ^26^ ^]^ and revealed an efficient degradation capacity of the organic molecules, namely methyl orange (MO) and paracetamol. However, the cellulose membrane is not as robust and stable as our silica‐based porous MSs, which might limit the recyclability of the photocatalytic system.

Contrary to most of the reported support materials, our MSs exhibit meso‐ and macroporosity, which favor the light‐harvesting and shorten the mass transfer/diffusion tracks, that is, increase the capacity for mass transport and promote the contact between pollutants and photocatalyst. Some of the above‐reported strategies may also exhibit issues in terms, for example, of chemical inertness or safe environmental disposal after use.

Aeroxide P25 commercial TiO_2_ nanoparticles (Evonik) are by far the most used in the literature for supported photocatalytic experiments.^[^ ^17^, ^18^, ^21^, ^22^, ^24^, ^25^, ^28^, ^29^ ^]^ However, in some cases, in situ synthesized NPs, through the respective organic precursors, are also used.^[^ ^16^, ^19^ ^]^

Finally, regarding the type of solar reactor, the slurry (batch) reactors are, by far, the most common ones found in the literature. Continuous flow reactors are typically not as efficient as batch reactors in terms of photocatalytic degradation, which might be explained by the lower residence time, that is, shorter contact between active species and pollutant species in the flow‐through apparatus. However, continuous set‐ups are much more aligned to a real application, involving real wastewaters in pilot plants, because larger volumes are treated in a continuous fashion, requiring less iterations and maintenance operations.

This paper reports the development of photocatalytic microspheres through the immobilization of Trizma functionalized TiO_2_ NPs within SiO_2_ MSs with interconnected macroporosity, which will be assessed for the degradation of MO and paracetamol, under simulated solar illumination. It involves much more than a conventional photocatalytic system for water purification, which typically relies on photocatalytic NPs dispersed in the liquid reaction medium (slurry reactor, batch process) and time‐consuming and complex nanofiltration process for recovery of the catalysts. Instead, it is about a continuous flow‐through operation and enough robustness and stability for handling and reutilization, due to the prevalence of heterogeneous photocatalysis (not adsorption) phenomenon and the chemical and mechanical resistance of the employed MSs. This new technology targets solar reactors tailored for efficient solar light harvesting and economically viable wastewater purification schemes. It has a strong application component and envisages a wide environmental impact in the field of water quality and security. The target of this technology is not only for developing countries, but also for developed countries, especially in what regards wastewater decontamination in industrial processes.

## 2 Experimental Section

### 2.1 Materials

Titanium (IV) tetrachloride (99.9% trace metals basis), benzyl alcohol (puriss., 99–100.5% (GC)), 2‐amino‐2‐(hydroxymethyl)‐1,3‐propanediol (Trizma base, puriss., ≥99.7%), chloroform (≥99.8%), diethyl ether (for HPLC, ≥99.9%, inhibitor‐free), ethanol (absolute), tetraethylorthosilicate (TEOS, 98%), methyl orange (MO), and acetaminophen (paracetamol, ≥99%) were purchased from Sigma‐Aldrich. Hydrochloric acid 37% was obtained from Carlo Erba. Decahydronaphthalene (decalin, a mixture of *cis* and t*rans* isomers, 98%) and sorbitan monooleate surfactant (Span 80, HLB: 4.3) were obtained from Merck. 3‐Glycidyloxypropyl)trimethoxysilane (GPTMS, Xiameter OFS‐6040, >98.5%) was supplied by Dow. All chemicals were used without further purification.

### 2.2 Synthesis of Trizma‐Functionalized TiO_2_ Nanoparticles (NPs)

2‐amino‐2‐(hydroxymethyl)‐1,3‐propanediol ((HOCH_2_)_3_CNH_2_ – Trizma) functionalized TiO_2_ nanoparticles were synthesized following an upscaled and modified route developed by Niederberger et al.^[^ ^10^ ^]^ 3.4 mmol (414 mg) of Trizma were dissolved in 90 mL benzyl alcohol at 80 °C in an oil bath under vigorous stirring. After letting the solution cool down to room temperature, 40.9 mmol (4.5 mL) of titanium(IV) chloride were added. The reaction solution was again heated to 80 °C and left there for 24 h. The white precipitate was collected by centrifugation and washed three times with chloroform and three times with diethyl ether. 30 mL of deionized water were added to the wet powder. After stirring the sol overnight, vacuum was applied to remove the residual diethyl ether. Deionized water was added to the NPs dispersion to reach a 62.5 mg mL^−1^ concentration. The TiO_2_ NPs concentration was determined by placing an accurate volume of the aqueous dispersion in a quartz vial, using a calibrated pipette, and weighing before and after drying at 60 °C for 48 h. The resulting dried NPs were then subjected to different heat treatments to assess their crystalline features, chemical structure, and organic content.

### 2.3 Synthesis of the Silica‐Based Microscaffolds (SiO_2_ MSs)

Silica‐based microscaffolds were synthesized according to a modified method previously reported,^[^ ^14^ ^]^ developed by Marques et al. This process follows the steps of any silane‐based sol–gel reaction. However, condensation reactions are made to occur inside the water droplets of a templating water‐in‐oil (W/O) emulsion, to obtain particles with a spherical shape. To achieve the desired continuous, interconnected macroporosity within the microspheres, we selected the synthesis parameters to assure that the freezing of the structure occurs coincidently with the critical point for phase separation by spinodal decomposition.

First, 18.7 mL of GPTMS (Xiameter OFS‐6040) and 21.4 mL of TEOS were mixed at a molar ratio of 1:0.89, in a closed container under magnetic stirring with 15 mL of HCl 0.05 m aqueous solution, for 50 min. Then, 300 µL of HCl (37%) was added, and the mixture was stirred for an additional 10 min.

Separately, the W/O emulsion was prepared by mixing 45.0 mL of distilled water, 113.6 mL of decalin, and 6 g of Span 80 at 18 000 rpm using a dispersing instrument (IKA T18 digital ULTRA‐TURRAX, Staufen, Germany) for 10 min.

The emulsion and the prehydrolyzed silanes solution were then added to the reactor and kept at a stirring speed of 600 rpm for a selected time/temperature protocol, involving a temperature rise of 5 °C every hour from 80 to 95 °C.

In the end, the microscaffolds were washed with acetone through vacuum filtration and dried overnight at 45 °C.

### 2.4 Preparation of the Photocatalytic Microspheres: TiO_2_ NPs Loaded SiO_2_ MSs

**Figure 1:**
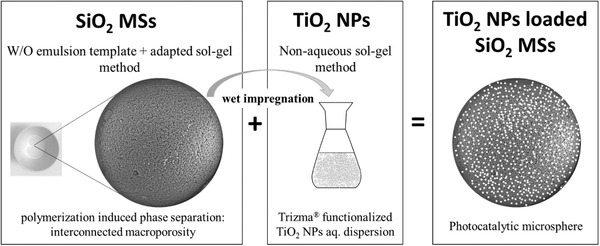
Approach for the synthesis of the photocatalytic microspheres.

The photocatalytic microspheres, consisting of TiO_2_ NPs loaded SiO_2_ MSs, were prepared by direct wet impregnation with an aqueous dispersion of TiO_2_ NPs. This process resulted in the immobilization of TiO_2_ NPs on the MSs’ pores surface, promoted by solvent evaporation and electrostatic interactions (**Figure** **1**).

First, the SiO_2_ MSs were heat‐treated for 30 min at 900 °C, and 372 mg of the resulting sample was immersed in 2.1 mL of TiO_2_ NPs aqueous dispersion (62.5 mg mL^−1^) placed in a glass vial, and subjected to sonication for 2 min (37 Hz / 100 power). Then the glass vial with the mixture was placed in an oven at 60 °C for 15 h to promote solvent evaporation. Dried whitish small granules (powder) were obtained, which were subjected to heat treatment (1 h at 500 °C). Weighing the powder (TiO_2_ NPs loaded SiO_2_ MSs) after the heat treatment at 500 °C, an increment by ≈22% of the MSs weight was obtained, which suggested a loading of 22 wt% with TiO_2_ NPs. This value was comparable with the theoretical value of 26 wt% of TiO_2_ NPs loading. It was expected that some loss of NPs might occur during the different steps of the immersion, drying, and heat treatment procedure.

After heat treatment, the sample was washed with 20 mL of distilled water (by vacuum filtration), and the resulting washing water was collected for analysis to check the effectiveness of the grafting process.

The resulting photocatalytic microspheres were then characterized.

### 2.5 Characterization of the TiO_2_ NPs and Loaded and Unloaded MSs

Scanning electron microscopy (SEM) images were achieved on a Zeiss Leo‐1530, aimed at assessing the morphology, size, and porosity of the loaded and unloaded MSs. The MSs’ size distribution was obtained through SEM images by measuring the diameter of 100 MSs using the Fiji software.^[^ ^30^ ^]^

A few MSs were broken on purpose to detect the presence of TiO_2_ NPs on their internal pores’ surface. The samples were sputtered with a 3 nm thin platinum layer before SEM imaging. Energy dispersive spectroscopy (EDS) was carried out to compare the elemental composition of the loaded MSs before and after the photocatalytic tests. Transmission electron microscopy (TEM) and high‐resolution transmission electron microscopy (HRTEM) were carried out on an FEI Talos F 200X operated at 200 kV, in both TEM and scanning (STEM) modes. A well‐diluted dispersion of TiO_2_ NPs in ethanol was placed on a Cu grid for such purpose. STEM analyses were performed with a high angle annular dark field detector (HAADF STEM).

To study the crystalline nature of the MSs and NPs, powder X‐ray diffraction (XRD) patterns were recorded. For such purpose, a PANalytical Empyrean diffractometer equipped with a PIXcel 1D detector was employed in reflection mode, using Cu Kα X‐ray irradiation and operating at 45 kV and 30 mA. The samples were crushed in an agate mortar and pressed into the sample holder to achieve a flat layer, before the mounting on XRD equipment.

Attenuated total reflectance Fourier transform infrared spectroscopy (FTIR‐ATR) measurements were performed on a Bruker Alpha FT‐IR spectrometer equipped with a mid‐infrared source and a diamond ATR optics, to study the chemical structure of the loaded and unloaded MSs.

Nitrogen sorption experiments were performed on a Quantachrome Autosorb‐iQ‐C‐XR at 77 K. Samples were outgassed at 100 °C for at least 24 h, before the gas sorption analyses. The specific surface area of the TiO_2_ NPs and loaded and unloaded MSs was determined via the Brunauer–Emmett–Teller (BET) method. The pore size and pore volume were determined by a density functional theory (DFT) analysis using a non local DFT (NLDFT) calculation model for nitrogen at 77 K, assuming cylindrical pores in silica.^[^ ^31^ ^]^

Thermogravimetric analysis (TGA) was carried out using a Mettler Toledo TGA/SDTA instrument under an air atmosphere at a heating rate of 10 °C min^−1^ and an airflow of 50 mL min^−1^. It was employed to assess the elimination of organic groups and corroborate FTIR analyses.

Hg porosimetry was performed to assess porosity on the MSs, using an Autopore IV 9500 from Micromeritics, and a constant contact angle of 140°. The samples were heated to 120 °C for 2h before measurements.

UV–vis spectroscopy measurements were performed with a JASCO V‐770 instrument, in the region of 200–800 nm, on the aqueous dispersions containing synthesized TiO2 NPs (0.1 mg mL^−1^) and on the washing waters taken previously and after the MSs loading process. UV–vis diffuse reflectance spectrum (DRS) of the NPs and loaded MSs was recorded as well.

### 2.6 Photocatalytic Studies

**Figure 2:**
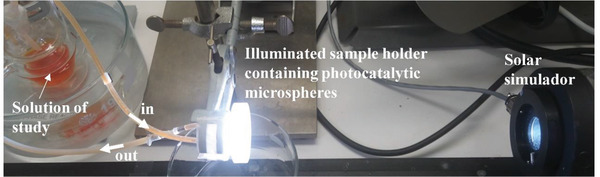
Photograph of the experimental set‐up (continuous flow) used for the photocatalytic tests.

The photocatalytic activity of the TiO_2_ NPs loaded SiO_2_ MSs (photocatalytic microspheres) was tested through the decomposition of MO and paracetamol under solar illumination, at 25 °C and at a pH of 7, which is the closest to a real panorama. A continuous flow reactor and the set‐up of **Figure** **2** were employed, which is the same as reported in ref. ^[^ ^26^ ^]^.

**Figure 3:**
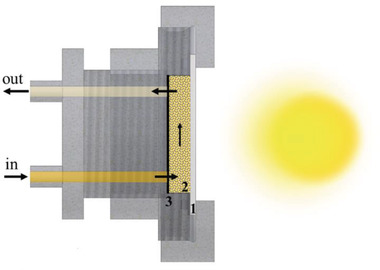
Schematic representation of the illuminated sample holder: 1‐ quartz window to allow exposure to solar light; 2‐ chamber (holder) filled with photocatalytic microspheres; 3‐ filter to ensure that there are no microspheres released with the flow. The inlet and outlet Teflon tubes allow the continuous flow of the pollutant through the microspheres.

The photocatalytic microspheres (≈300 mg) were placed in a custom made PMMA cell chamber^[^ ^26^ ^]^ (**Figure** **3**) and slightly pressed against a quartz window (1, in Figure 3), onto which the solar irradiation impinged during the tests. The sample holder consisted of the cell and quartz window, which are two movable parts screwed on a central body.

The cell was connected to a peristaltic pump (Ismatec, Wertheim, Germany) and a reservoir with Teflon tubes. The reservoir was filled with 100 mL of a 20 ppm solution (MO or paracetamol), and the flow set by the pump was 5 mL min^−1^. The solution to treat was then allowed to flow through the compacted microspheres (≈1 mm thick layer of microspheres, 2 in Figure 3) placed inside the chamber. A filter (3 in Figure 3) placed in between the microspheres and the cell cover where the Teflon tubes were connected ensured that all microspheres were kept inside the chamber, and no unwanted release of microspheres occurred with the flow.

First, the solution was made to flow through the microspheres in the dark for 30 min, and the concentration was checked regularly. Then, the decomposition experiments were started by turning‐on solar illumination. It is provided by a solar simulator (model 67 005) from Newport Spectra‐Physics, Darmstadt, Germany, equipped with an Arc Lamp Power Supply (model 69 911) and a 300 W Xe lamp operated at 255 W. The intensity was adjusted to 1000 W m^−2^ (equivalent to 1 sun) by establishing the correct distance between the cell and the lamp. The concentration of the pollutant (MO or paracetamol) was checked at different periods through UV–vis spectroscopy (absorption spectra) by extraction of 1 mL aliquots that were discarded after analysis. The absorbance was converted to the pollutant's concentration through a calibration curve (Figures S1 and S2, Supporting Information) in the used concentration range, which was fitted with a linear equation. For the photocatalytic efficiency assessment, the value of 22 wt% of TiO_2_ NPs loading, obtained by weighing, was employed.

## 3 Results and Discussion

### 3.1 Characterization of the TiO_2_ NPs

The as‐synthesized dried TiO_2_ NPs were subjected to different temperatures to optimize the heat treatment to be employed after the wet impregnation process in the photocatalytic microspheres (TiO_2_ NPs loaded SiO_2_ MSs) preparation. The selection of the best heat treatment, namely 60 min at 500 °C, was made in terms of the best compromise among small NP size, anatase crystalline nature, and low‐organics composition.

**Figure 4:**
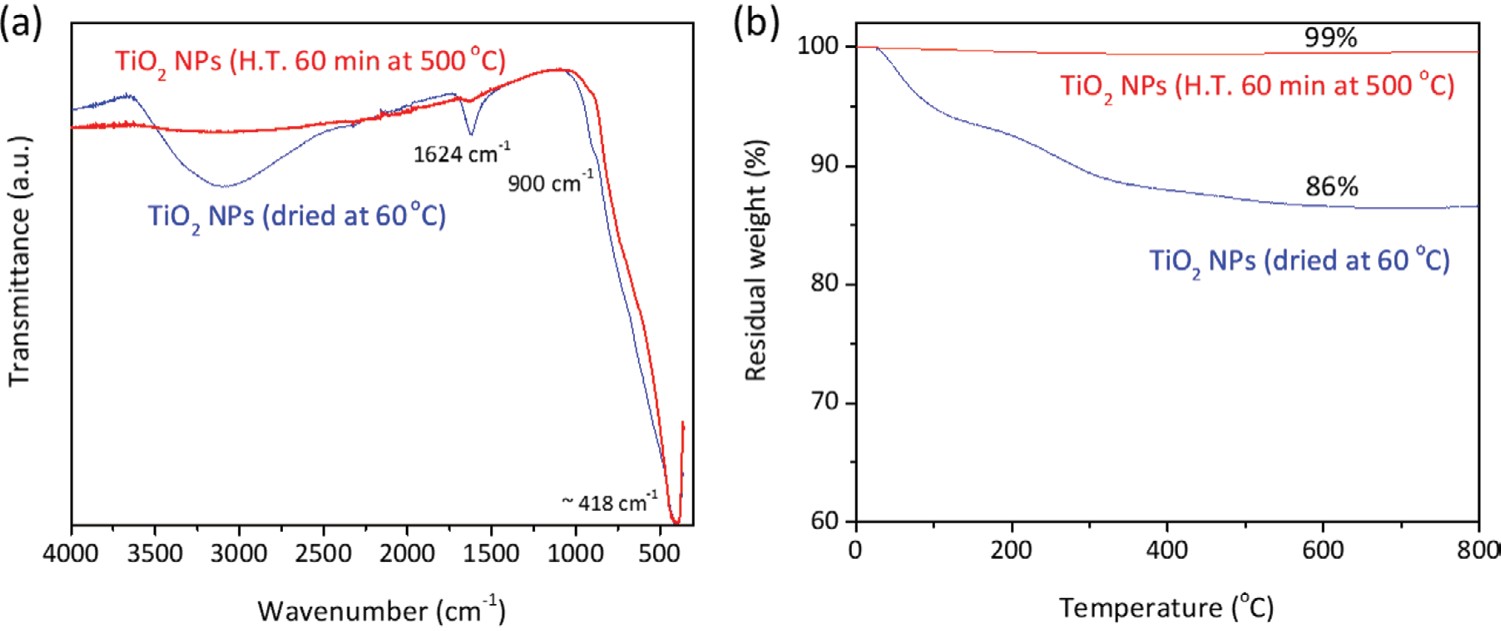
a) FTIR‐ATR spectra and b) thermogravimetric curves of the TiO_2_ NPs dried at 60 °C and heat‐treated for 1 h at 500 °C.

FTIR‐ATR spectroscopy analysis on dried and heat‐treated TiO_2_ NPs was performed to assess the chemical structure on the surface of the NPs (**Figure** **4**a). A strong and broad absorption peak at ≈418 cm^−1^ is the main feature for both samples, dried and heat‐treated, which reveals the presence of Ti—O—Ti linkages (Ti—O bending mode) from TiO_2_ composition. Absorption in the —OH stretching region (3500–3000 cm^−1^) is another main spectral feature for the dried sample, as well as the δ(H—O—H) bending at 1624 cm^−1^. These latter peaks, ascribed to surface adsorbed OH groups or water molecules, basically disappear with the heat treatment at 500 °C. A large shoulder on the high‐frequency side of the 418 cm^−1^ peak, at ≈900 cm^−1^, is also found to be present in the dried sample's spectrum. It might be related to bending vibrations of the C—H bonds of the aromatic groups, from residual benzyl alcohol employed in the synthesis of the TiO_2_ NPs. These bonds can also contribute to the wide absorption band at 3100–3030 cm^−1^.

Additionally, Trizma functionalization of the NPs also might contribute with O—H, N—H, and C—H stretching at 3500–3000, 3300–3000, and 3000–2850 cm^−1^, respectively. The N—H bending and wagging vibrations of the primary amine from Trizma are found in the regions 1650–1580 and 910–665 cm^−1^, respectively. They are of higher intensity in the dried sample than in the heat‐treated one, which reveals that Trizma might be present solely at a residual level if not eliminated at all upon the heat treatment at 500 °C. It should be stressed that the strategy of synthesizing Trizma functionalized TiO_2_ NPs was twofold: 1) the presence of amine groups is responsible for a more positively charged TiO_2_ surface, which favors the immobilization of TiO_2_ NPs onto the SiO_2_ MSs (negatively charged) by electrostatic interactions; 2) these NPs tend to self‐assemble into a 3D network with large pores, which is a consequence of the oriented attachment process that the NPs undergo upon selective removal of the Trizma stabilizer from the {001} facets when soaked in hot water.^[^ ^32^ ^]^ This fact potentially enables the formation of a more porous layer of TiO_2_ NPs onto the pores’ surface of the SiO_2_ MSs, which facilitates the contact between the active species and the pollutant molecules and therefore, leads to a higher photocatalytic activity.

The presence of Trizma is, therefore, important at the wet impregnation (loading) stage of the MSs. However, after the loading process, Trizma can be eliminated via heat treatment, to target fully inorganic photocatalytic microspheres. These MSs are potentially more mechanically, chemically, and thermally stable, that is, more durable, enabling many reusing cycles of the photocatalytic system. Moreover, the TiO_2_ NPs synthesized in this work (Trizma functionalized) and heat‐treated at 500 °C for 60 min, exhibited a better performance in terms of absorbance (redshift) at wavelengths up to 450 nm, than the commercially available TiO_2_ NPs (Aeroxide P25), as depicted in Figure S3, Supporting Information. Such redshift may indicate the lowering of the bandgap energy of TiO_2_, desired for solar‐driven photocatalysis.

Thermogravimetric analysis (Figure 4b) revealed that ≈14 wt% of the dried TiO_2_ NPs corresponds to organic matter and moisture. That value is found to tend to zero with the heat treatment at 500 °C, indicating the inorganic nature of the heat‐treated NPs.

**Figure 5:**
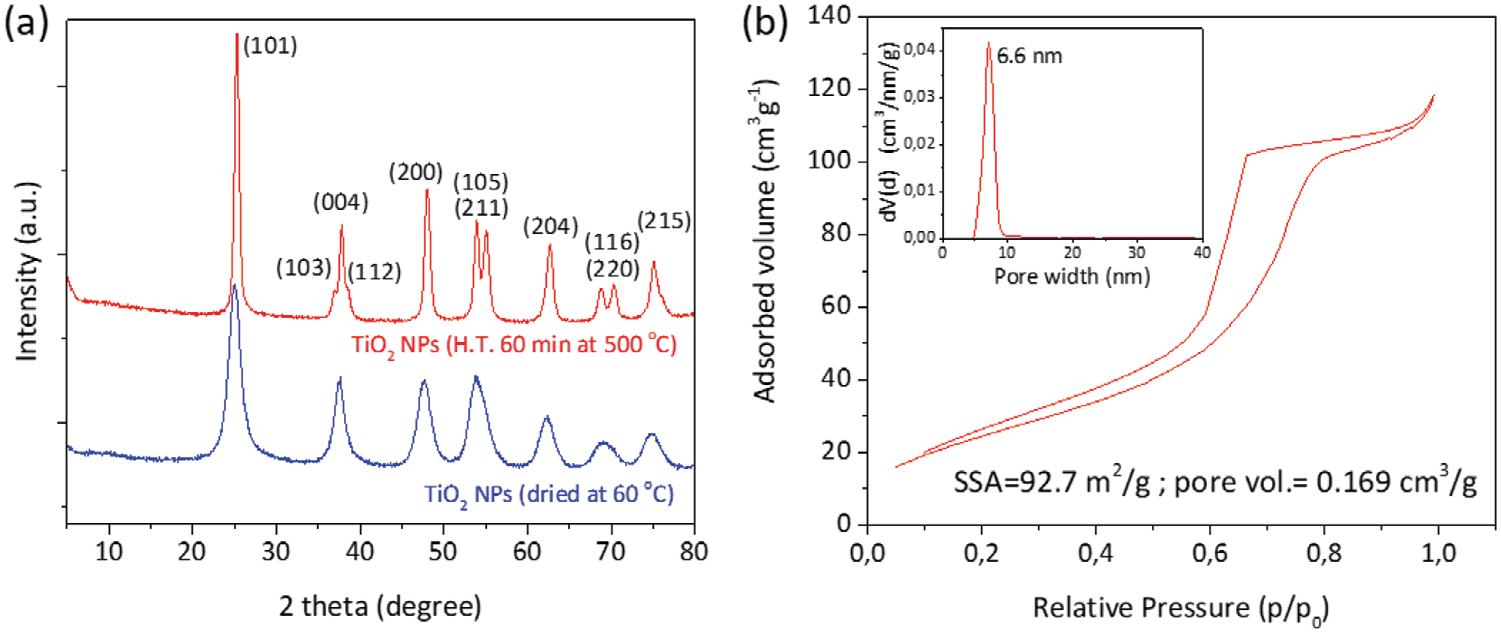
a) Powder XRD patterns of as‐synthesized dried TiO_2_ NPs and heat‐treated TiO_2_ NPs. The diffraction peaks are indexed according to the anatase TiO_2_ phase. b) BET isotherm of heat‐treated TiO_2_ NPs. The inset shows the mesopore size distribution, peaked at 6.6 nm.

Powder XRD allows us to identify the particles as highly crystalline anatase TiO_2_ (01‐084‐1285), even the as‐synthesized dried NPs (**Figure** **5**a), which enlightens the potential of the employed non‐aqueous sol–gel method to achieve crystalline NPs without the need for heat treatment. No other crystalline titanium oxide polymorph, besides anatase, was detected. Crystallites smaller than ≈120 nm create broadening of diffraction peaks, which can be used to quantify the average crystallite size of NPs using the Scherrer equation (Equation (1))

(1) $$\begin{array}{c} D\;=\frac{K\;\lambda }{\beta \;\cos\theta } \end{array}$$

where *D* is the average size of the crystals, *K* is the Scherrer constant (0.9 in this work), λ is the wavelength of the incident radiation (1.5418 Å), β is the full width at half maximum (radians) of the diffraction peak and θ is the Bragg's angle. All diffraction peaks, in Figure 5a, are relatively broad due to the nanosize of the crystals. The average crystallite size of the TiO_2_ NPs was estimated from the most intense diffraction peak (101) at 2θ angle of 25.2°, to be about 5 nm for the dried NPs, and 13 nm for the heat‐treated NPs. It can be concluded that the treatment at 500 °C only affects the growth of the NPs and not their crystalline phase. The same sample of heat‐treated TiO_2_ NPs was analyzed by N_2_ physisorption (Figure 5b), showing a specific surface area (SSA) of 92.7 m^2^ g^−1^ and a “pore width”, that is, space between particles, peaked at 6.6 nm, ranging from 5 to 10 nm.

**Figure 6:**
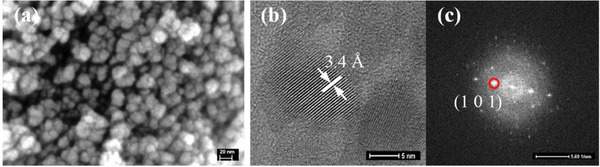
a) SEM image (scale bar: 20 nm) b) HRTEM image (scale bar: 5 nm) and c) corresponding selected area electron diffractogram (scale bar: 5 nm^−1^) of TiO_2_ NPs heat‐treated for 1 h at 500 °C.

The morphology of the heat‐treated TiO_2_ NPs was assessed by SEM. **Figure** **6**a shows the tendency of the TiO_2_ NPs to aggregate in small clusters. This reveals that strategies to control their distribution are critical, such as the one of the present work, based on the use of scaffolds with interconnected porosity to enable their distribution and grafting along the surface.

The characterization of the TiO_2_ NPs was complemented by HRTEM, which reveals that these NPs are crystalline. Figure 6b shows well defined crystallographic planes with an interplanar distance of 3.4 Å, which corresponds to that of the plane (101) of anatase crystals (Figure 6c). These findings corroborate the results from powder XRD (Figure 5a), and it can be concluded that the employed non‐aqueous method was successful for obtaining good control over crystallinity, size, and surface composition of the NPs to be used in the loading of the SiO_2_ MSs.

### 3.2 Characterization of the Unloaded and TiO_2_ NPs Loaded SiO_2_ MSs

**Figure 7:**
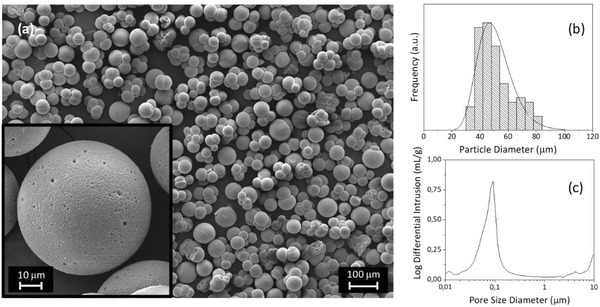
Unloaded SiO_2_ MSs used as scaffolds for TiO_2_ NPs loading: a) SEM image of the unloaded SiO_2_ MSs (scale bar: 100 µm), where the inset shows one MS at a higher level of magnification (scale bar: 10 µm). b) Particle size distribution. c) Pore size distribution curve.

The synthesis process of the SiO_2_ MSs employed in this work consists of a new approach based on W/O emulsification combined with an adapted sol–gel technique. It has been recently described elsewhere,^[^ ^14^ ^]^ including the study of the reaction medium evolution during the synthesis of the MSs. Specific synthesis reaction parameters were selected, such as surfactant amount, emulsification speed, pre‐hydrolysis time, and HCl/silanes molar ratio, to achieve MSs with the desired particle size distribution and interconnected porosity. The obtained MSs of spherical shape and porous morphology, are represented in the images of **Figure** **7**a. They display a diameter (peak maximum mode) of 47 µm (monomodal lognormal distribution), as shown in Figure 7b. The Hg porosimetry analysis (Figure 7c) of the unloaded MSs revealed a pore diameter peaked at ≈100 nm, large enough for the loading with the TiO_2_ NPs, and a macroporosity of 64%. These features allow a large extent of NPs loading, but also ensure that the photocatalytic microspheres will be robust enough to be handled and reused many times.

**Figure 8:**
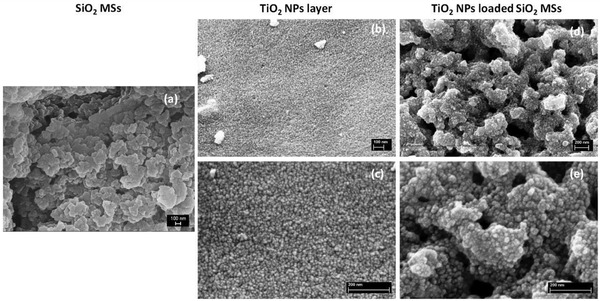
SEM images of the silica scaffolds (a), TiO_2_ NPs layer deposited on a flat surface (b,c) and TiO_2_ NPs loaded scaffolds (d,e). Scale bar: 100 nm (a,b); Scale bar: 200 nm (c–e).

The loading of the SiO_2_ MSs with Trizma functionalized TiO_2_ NPs, by direct wet impregnation, was evaluated by SEM. **Figure** **8**a shows a magnification of the pristine silica scaffolds, exhibiting the desired interconnected macroporosity, which is present in the whole particle, not only at the surface. In contrast, Figure 8d,e shows the same structure covered with a continuous layer of NPs. The NPs immobilized within the scaffolds appear to exhibit similar size and morphology than NPs deposited on a flat surface (Figure 8b,c) subject to the same heat treatment. They successfully cover the internal pores’ surface of the microspheres along the MSs’ entire cross‐section, while maintaining their characteristic interconnected porosity, critical to enable an easy flow of the water through the microspheres during the photocatalytic tests in the liquid phase.

It should be noted that SEM‐EDS revealed the presence of 16.7 wt% of Ti, however, the value for TiO_2_ NPs, obtained by weighing was 22 wt%, as referred in Section 2.

**Figure 9:**
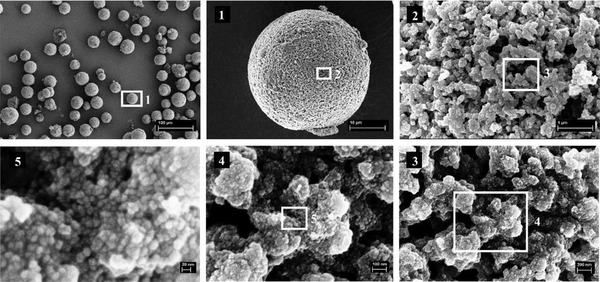
SEM images of SiO_2_ MSs after impregnation with TiO_2_ NPs. Scale bar is, by increasing order of magnification: 100 µm, 10 µm, 1 µm, 200 nm, 100 nm, and 20 nm.

**Figure** **9** shows a series of SEM images of the loaded MSs (photocatalytic microspheres) at different magnifications, which evidences that the loading process neither affects the shape and physical integrity of the SiO_2_ MSs, nor their inherent macroporosity. The only difference, when comparing with the unloaded MSs, is the presence of a thin layer of NPs all over the porous surface.

**Figure 10:**
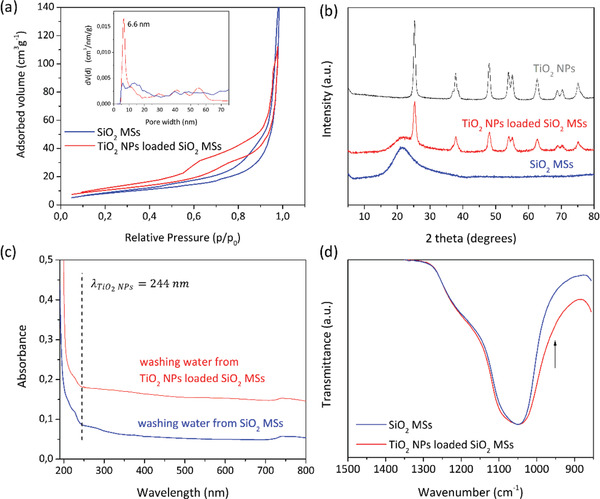
a) N_2_ adsorption–desorption isotherms and pore size distribution (inset) and b) XRD diffractograms for unloaded and loaded SiO_2_ MSs. c) Absorbance spectra of the washing water from the unloaded and loaded MSs. d) Normalized ATR‐FTIR spectra in the Si‐O‐Si region of the spectra.

Loading of the MSs with TiO_2_ NPs results in an increase (by 26%) of the MSs’ SSA from ≈31 to 42 m^2^ g^−1^, and in the appearance of mesoporosity peaked at 6.6 nm due to the layer of TiO_2_ NPs formed on the MSs’ surface, as shown in **Figure** **10**a. The higher surface area of the photocatalytic microspheres is useful for better contact between the pollutant species and the active sites and enables size and shape selectivity. Additionally, the presence of anatase TiO_2_ NPs is detected on the loaded MSs by XRD (Figure 10b). The crystallographic phase and crystal size are the same as in individual TiO_2_ NPs subject to the same heat treatment (1 h at 500 °C). On the other hand, SiO_2_ scaffolds, previously heat‐treated at 900 °C, appear to be in the amorphous state, and according to the thermogravimetric analysis, they are inorganic, without any weight loss up to 900 °C (Figure S4, Supporting Information). Figure 10c shows the absorbance spectra of the water that resulted from the washing step carried out on the unloaded and loaded MSs, that is, before and after the impregnation process of TiO_2_ NPs. There is no absorbance at the characteristic wavelength of TiO_2_ NPs (244 nm). So, we can conclude that no significant release of TiO_2_ NPs into the surrounding medium occurs, when performing washing by vacuum‐assisted filtration (high cross‐flow). This observation confirms the efficient immobilization and also the robust grafting of the NPs onto the SiO_2_ surface. Favored by the heat treatment performed at 500 °C, the TiO_2_ NPs might be bound to the SiO_2_ MSs via condensation reaction between the surface hydroxyl group of TiO_2_ (titanols, Ti—OH) and silanols (Si—OH) from the MSs. Further evidence for this fact would be the appearance/increase of the FTIR peak at ≈945 cm^−1^ related to Si‐O^−^, where the establishment of Si—O—Ti bonds (from the grafting of the NPs) play a role. However, this feature is not clear because the effect of the strong, broad peak at ≈418 cm^−1^, from the presence of TiO_2_ NPs, slightly masks this region (Figure 10d).

### 3.3 Photocatalytic Activity of the TiO_2_ NPs Loaded SiO_2_ MSs for Methyl Orange and Paracetamol Degradation

MO is a carcinogenic water‐soluble azo dye, widely used in the textile industry, manufacturing printing paper processes, etc. It is stable and exhibits low biodegradability and, hence, it is difficult to be removed from aqueous solutions by conventional water treatment/purification methods.^[^ ^33^ ^]^ The photocatalytic activity of the TiO_2_ NPs loaded SiO_2_ MSs was investigated by following the solar‐induced degradation kinetics of aqueous MO dye solutions, as a proof of concept. The UV–vis absorbance spectra of aqueous MO solutions have two distinct absorption peaks appearing at 271 and 463 nm, whose intensity decreases linearly with the decrease of the MO concentration, see calibration curves for MO (Figure S1, Supporting Information). The photocatalytic degradation kinetics of MO, using the continuous flow‐through reactor shown in Figure 2 with the customized sample holder displayed in Figure 3, was studied. The absorbance (colorimetry) of 1 mL aliquots of the MO solution to treat, withdrawn from the reservoir at selected time intervals, was checked, which gives the concentration of the dye.

**Figure 11:**
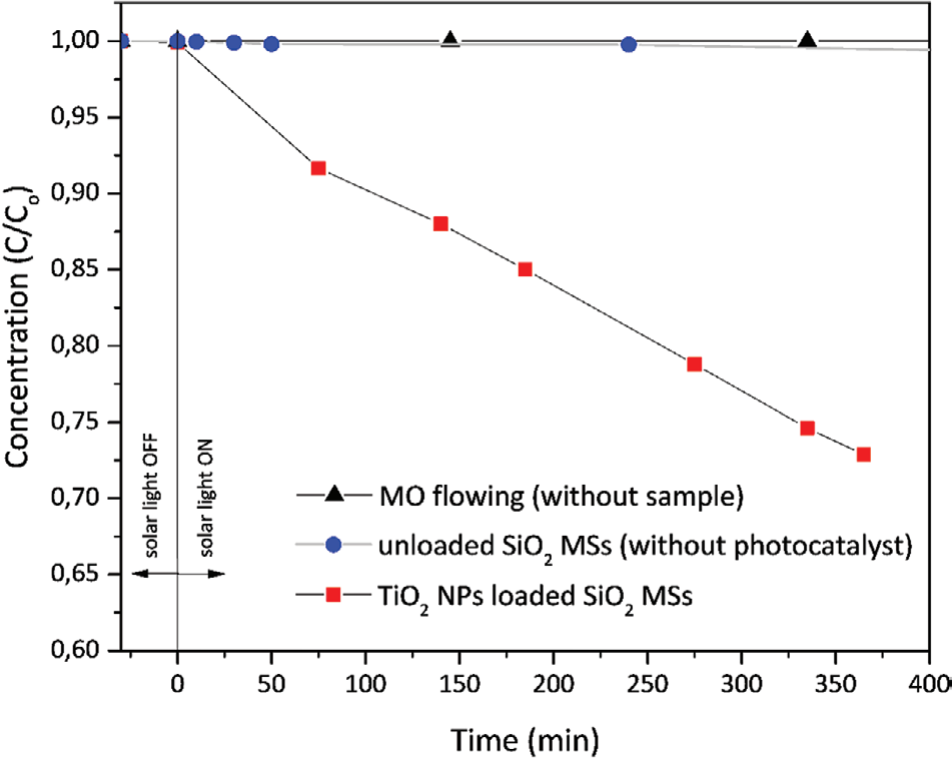
Plots of *C*/*C*_o_ versus time for the unloaded and TiO_2_ NPs loaded SiO_2_ MSs. MO shows no photodegradation at all during the experiment time, in the absence of photocatalyst. Experimental conditions: 25 °C, pH = 7, mass_(TiO2 NPs)_/mass_(MO)_ = 25, 100 mL of 20 ppm MO aq. solution, 300 mg of TiO_2_ NPs loaded SiO_2_ MSs (66 mg TiO_2_), flow =  5 mL min^−1^, irradiance = 1000 W m^−2^ (1 sun).

At neutral pH, TiO_2_ surface is close to its isoelectric point, but already negatively charged,^[^ ^34^ ^]^ so that MO, an anionic dye, is not expected to significantly adsorb on the TiO_2_ NPs that cover the SiO_2_ MSs. In any case, this phenomenon was assessed. Right after mounting the experiment, by placing the sample (microspheres) inside the chamber, the constant flow (5 mL min^−1^) with the MO solution to treat is started and maintained without solar illumination for 30 min. This procedure was followed to check if degradation in the dark occurs. This period was considered to equilibrate the system and ensure that no relevant adsorption of MO occurs on the microspheres since no variation of the MO concentration was observed. Tests through light‐dark cycles were carried out to check for this phenomenon also during the experiment (Figure S5, Supporting Information). Photocatalytic degradation did not occur in the dark, even in the presence of the photocatalyst (loaded MSs). Also, no photocatalytic activity was shown by the unloaded SiO_2_ MSs, with the Xe lamp switched on, during more than 400 min, as indicated in **Figure** **11** (blue circles).

However, when performing the test with the TiO_2_ NPs loaded SiO_2_ MSs, a significant decrease of the MO peak intensity was observed with the exposure to the simulated solar light (Figure 11, red squares), with ≈30% of the organic dye being decomposed within 375 min.

This experiment reveals that the loaded microspheres are potential photocatalytic systems for in‐flow, continuous, water purification processes. The mechanical strength and chemical/thermal stability of SiO_2_ material and the efficient photocatalyst (TiO_2_ NPs) immobilization, enable frequent reuse of this system.

It should be noted that a concentration of pollutant solution of 20 ppm has been chosen for comparative reasons, that is, a concentration of 20 ppm or less was employed in most of the studies to which our results are being compared in this paper. Considering the same volume of pollutant solution, the photocatalytic activity is expected to be higher for a lower pollutant concentration, since photocatalysis is a heterogeneous catalysis phenomenon, where the catalyst surface availability plays an important role in the catalytic activity. Regarding the flow of the pollutant solution (5 mL min^−1^), the larger the flow, the larger the pollutant quantity flowing through the photocatalyst. At the same time, there should be enough time for the pollutant to interact with the photocatalyst and to react. The photocatalytic reaction limits the flow rate, so that the best compromise achieved for the employed set‐up in this experiment was 5 mL min^−1^.

The plot of *C*/*C*_o_ versus time for degradation of MO is shown in Figure 11. A linear relationship was observed between the degradation rate of MO and irradiation time, so that degradation of the pollutant molecules was assumed to follow pseudo‐first‐order kinetics. The apparent rate constant (*k*) was computed using Equation (2), where *C* is the pollutant concentration at a given time *t*, giving a value of 0.0009 min^−1^ for MO degradation by TiO_2_ NPs loaded SiO_2_ MSs.

(2) $$\begin{array}{c} \ln\frac{C}{C_{o}}=\;-kt \end{array}$$

The direct comparison of degradation efficiency, namely the achieved *k* with other works in the literature, is not straightforward, because it does depend on various factors, such as: i) concentration of TiO_2_ NPs employed in the tests; ii) the photocatalyst/pollutant weight ratio; iii) the radiation source and irradiance; and iv) the set‐up used in the photocatalysis test, etc. Reaction conditions are often different, and unfortunately, some published works tend to lack essential parameters which are critical in the concerted comparative analysis among various studies.

**Table** **1** lists the values for *k* (min^−1^) and TiO_2_ NPs weight normalized *k* (min^−1^ g^−1^), which enable to have an overall comparative assessment of the present study with some other works in the literature in a similar fashion to what has been done in ref. ^[^ ^26^ ^]^. It also allows us to conclude about the photocatalysis efficiency when TiO_2_ NPs are immobilized and when continuous flow reactors are employed versus batch reactors, which are strategies that favor upscaled and economically viable wastewater purification schemes.

**Table 1:** Experimental conditions and apparent rate constant (*k*) of the photocatalysis tests. Comparison of the present study with the literature, in what regards MO degradation by TiO_2_ anatase NPs

Photocatalyst	Pollutant	Photocatalyst/Pollutant weight ratio	Reactor type	Radiation source (irradiance)	*k* [min^−1^]	*k* [min^−1^ g^−1^]	Ref. (Year)
Synthesized TiO_2_ NPs (by non‐aqueous sol–gel) immobilized in macroporous SiO_2_ MSs (66 mg)	MO (20 ppm, 100 mL)	26	Continuous flow pH = 7	Simulated solar light (1 sun, 1000 W m^−2^)	0.0009	0.013	Present study
Synthesized TiO_2_ anatase (50 mg)	MO (7.5 mg L^−1^, 100 mL)	67	Batch pH = unknown	UV light	0.0028	0.057	^[^ ^42^ ^]^(2019)
Synthesized TiO_2_ anatase (100 mg)	MO (15 ppm, 100 mL)	52	Batch pH = unknown	Solar light	0.0048	0.048	^[^ ^43^ ^]^(2019)
Synthesized TiO_2_ NPs (by non‐aqueous sol–gel) immobilized in cellulose monolith (20 mg)	MO (20 ppm, 100 mL)	8	Continuous flow pH = 7	Simulated solar light (1 sun, 1000 W m^−2^)	0.0011	0.055	^[^ ^26^ ^]^(2018)
Synthesized TiO_2_ anatase (50 mg)	MO (20 mg L^−1^, 100 mL)	25	Batch pH = 4.8	Visible light (300W Xe arc lamp with cut‐off filter of 400 nm)	0.0016	0.003	^[^ ^44^ ^]^(2018)
TiO_2_ anatase NPs (1500 mg L^−1^)	MO (25 µmol L^−1^, 200 mL)	183	Batch pH = 6‐4	Simulated solar light (≈1116 W m^−2^)	0.0200	0.067	^[^ ^45^ ^]^(2015)
TiO_2_ anatase Aldrich (2000 mg)	MO (8 × 10^−5^ m aq. sol., 500 mL)	152	Batch pH = 7	Solar light (4325 Wh m^−2^)	0.0107	0.005	^[^ ^46^ ^]^(2002)

On the one hand, practical engineering applications of TiO_2_ photocatalysts require fixation on inorganic support materials to facilitate catalyst recovery. On the other hand, there is some controversy regarding the efficiency of reactors with immobilized photocatalysts. As an example, a significant decrease of photocatalytic degradation was reported for TiO_2_ immobilized in quartz sand and applied in a fixed‐bed configuration,^[^ ^35^, ^36^ ^]^ when compared to dispersed TiO_2_ (slurry).^[^ ^37^ ^]^ Such decrease might be due to part of the TiO_2_ NPs were not fully exposed to photon irradiation, possibly being in the shadow of the sand particles, or not entirely in contact with the pollutant species. However, suitable modification of the experimental conditions has enabled fast photocatalytic decomposition of pollutants in systems containing immobilized TiO_2_ particles.^[^ ^36^ ^]^ Photocatalytic activities of TiO_2_ NPs supported on silica or alumina,^[^ ^38^ ^]^ or sorbents such as clays,^[^ ^39^ ^]^ have also been reported to be lower than those for the corresponding TiO_2_ alone. Reduced crystallinity of the TiO_2_ NPs, when in combination with support materials, has been the reason for such an issue.^[^ ^40^ ^]^ However, the use of porous silicate materials or hydrophobic materials as scaffolds for TiO_2_ photocatalysts and optimized TiO_2_ crystallinity have been reported to result in more efficient photocatalytic systems.^[^ ^39^, ^40^, ^41^ ^]^ It should be stressed that in our work, TiO_2_ NPs employed in the immobilization process are already of controlled crystalline nature, namely TiO_2_ anatase, which does not change during the process of impregnation and post synthetic heat treatment.

By observing Table 1, the two works with higher reported values for *k* ^[^ ^45^, ^46^ ^]^ are, in fact, related to a much larger value of TiO_2_/MO weight ratio than our work (183 and 152 versus 26), and allow us to conclude that the TiO_2_ weight normalized *k*, calculated for our work (0.013 min^−1^ g^−1^), is close or even better than the one calculated for those two cases. Additionally, the work with a similar TiO_2_/MO weight ratio,^[^ ^44^ ^]^ of 25, exhibits a much lower value for the calculated TiO_2_ weight normalized *k* (0.003 min^−1^ g^−1^). It should be noted that a cut‐off filter of 400 nm was used in this case, limiting the relevant radiation to the vis range. Still, the experiment was carried out in acidic medium (pH = 4.8), which is known to favor the MO degradation, due to enhanced adsorption of MO on the surface of the photocatalyst. This improved absorption can be explained by electrostatic interactions between the positive photocatalyst surface and dye anions.^[^ ^47^ ^]^ Moreover, the other two works^[^ ^42^, ^43^ ^]^ that exhibit a relatively large calculated TiO_2_ weight normalized *k*, are indeed related to TiO_2_/MO weight ratios ≈5 times greater than the ones employed in the present study, that is, we use much less TiO_2_ NPs per mass of MO. It should be stressed that one of them^[^ ^42^ ^]^ makes use of UV radiation, which significantly increases the activity of the catalyst, while our results were obtained under simulated solar illumination.

All the works from the literature listed in Table 1 involve less appealing options based on batch‐type reactors and non‐immobilized TiO_2_ species, contrary to our work and ref. ^[^ ^26^ ^]^, which involve a continuous flow type of reactor and immobilized TiO_2_ NPs. Therefore, the photocatalytic microspheres developed in the present work offer a similar or better photocatalytic activity than state of the art, but with the advantage of being easily removed from the reaction medium. Finally, it should be stated that ref. ^[^ ^26^ ^]^ exhibits an impressive low value for the TiO_2_/MO weight ratio and a relatively high value for the calculated TiO_2_ weight normalized *k*. However, potentially not such a durable and robust photocatalytic system as the one of the present study, which is based on inorganic SiO_2_.

Paracetamol (*N*‐acetyl‐4‐amino‐phenol) is the most widely used human medicine and has been found at relatively high concentrations in treated wastewaters.^[^ ^48^, ^49^ ^]^ Despite its relatively low toxicity, it is a target for removal because of its continuous release in sewers, being a quasi‐persistent compound in the environment. Therefore, as another proof of concept, right after the end of the experiment with MO, the solution in the reservoir was replaced by 100 mL of 20 ppm of paracetamol, and a consecutive photocatalysis experiment was carried out, reusing precisely the same TiO_2_ NPs loaded SiO_2_ MSs that were located in the sample holder. This feasibility test was run overnight, after 30 min of exposure of the system to the dark with the flow at 5 mL min^−1^. 65% of paracetamol degradation was achieved after 14 h of simulated solar illumination (Figure S6, Supporting Information), which gives a *k* of 0.0013 min^−1^. This value is of the same order of magnitude when comparing with values in the literature. For instance, *k* of 0.0074 min^−1^ was achieved for the degradation of paracetamol, at pH = 5.5, with TiO_2_ P25, under sunlight.^[^ ^50^ ^]^ However, approximately the same value for the calculated TiO_2_ weight normalized *k*, of ≈0.02 min^−1^ g^−1^, was achieved for both cases. Again, it can be stressed that besides our system has the advantage of being easily removed from the reaction medium and is a continuous flow set‐up, our result for paracetamol degradation was obtained by consecutively reusing previously tested photocatalytic microspheres, without carrying out any specific washing treatment on the photocatalytic system.

### 3.4 Effect of the Photocatalytic Tests on the TiO_2_ NPs Loaded SiO_2_ MSs

**Figure 12:**
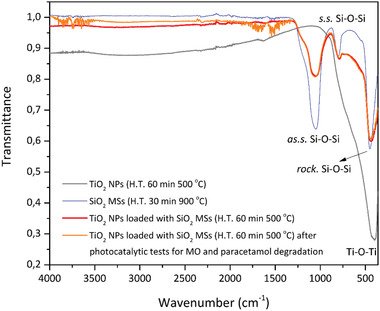
FTIR‐ATR spectra of TiO_2_ NPs, of unloaded SiO_2_ MSs and loaded SiO_2_ MSs before and after the photocatalytic tests.

The successful feasibility test performed for paracetamol degradation with reused photocatalytic microspheres (TiO_2_ NPs loaded SiO_2_ MSs) reveals their stability and capability to be reused without an intermediate washing step. **Figure** **12** shows the FTIR‐ATR spectra taken at different stages of the studies, namely those of individual TiO_2_ NPs and SiO_2_ MSs, and those acquired on the TiO_2_ NPs loaded SiO_2_ MSs before and after the photocatalytic tests for MO and paracetamol degradation.

After the heat treatment of the SiO_2_ MSs at 900 °C, broad and strong absorption bands peaked at ≈1060, 800 and 450 cm^−1^ are found in the spectrum revealing the presence of a silica‐based inorganic material, whose assignments are well explained in refs. ^[^ ^14^, ^51^ ^]^ for instance. It should be stressed that the band at ≈1060 cm^−1^ exhibits a large full width at half maximum (FWHM) and is slightly shifted to lower frequencies. This might be associated with the broadening of the bond angle distribution^[^ ^51^ ^]^ previously reported to derive from the large porosity characteristic of these MSs.^[^ ^14^ ^]^

Further evidence of porosity comes from the relatively strong shoulder of the peak at 1060 cm^−1^, located around 1200 cm^−1^, which has been associated with the presence of residual material porosity.^[^ ^52^ ^]^ With the loading process, the main feature arising at the FTIR‐ATR spectrum is the increased absorption in the range of 850–400 cm^−1^, which derives from the presence of TiO_2_ NPs. Also, the band at 450 cm^−1^ shifts to lower frequencies, and the *I*_450_/*I*_1060_ ratio becomes much more intense, revealing the success of the loading and immobilization process. It should be noted that the spectrum was obtained on the loaded sample, after the washing step. Finally, the loaded SiO_2_ MSs also exhibit a broad band (large FWHM) at 1060 cm^−1^, like the unloaded sample, which reveals that macroporosity does not significantly change with the loading process. The thin layer of NPs along the MSs surface does not affect their typical macroporosity, of critical importance for the mass transport and flow during the photocatalytic tests. This finding corroborates the SEM analyses done before and after the loading process (Figure 8).

Of relevance in Figure 12 is the substantial similarity of the spectra before and after the photocatalytic tests: they are perfectly superimposed between 1300 and 400 cm^−1^, and exhibit just some noise features in the range of 4000–1300 cm^−1^.

**Figure 13:**
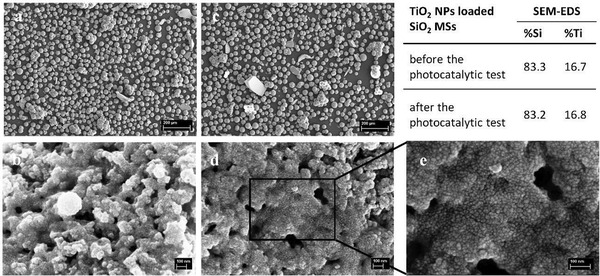
a,b) TiO_2_ NPs loaded SiO_2_ MSs before and c,d) after the photocatalytic tests (with MO and with paracetamol). Scale bar 200 µm (a,c); scale bar: 100 nm(b,d,e).

This finding suggests that the silica structure and the TiO_2_ NPs are not affected during the tests. This is further corroborated by the similar morphology of the photocatalytic microspheres, observed at the low and high magnification SEM images, as well as by the EDS analyses that reveal the same amount of Si and Ti before and after the photocatalytic tests (**Figure** **13**). We can, therefore, conclude that no significant amount of TiO_2_ is released from the photocatalytic microspheres into the medium during the photocatalytic tests.

## 4 Conclusions

The present paper reports the achievement of an efficient, low‐cost, and straightforward engineered photocatalytic system, driven by solar light, of high applicability in a real‐life scenario, targeting wastewater purification, that is, the degradation of organic pollutants. Controlled and stable crystalline anatase TiO_2_ NPs were synthesized at low temperature by a non‐aqueous benzyl alcohol sol–gel method, and immobilized within SiO_2_ microspheres with interconnected, continuous macroporosity, forming a well attached, thin layer of NPs along the pores’ surface. Electrostatic interactions and solvent evaporation phenomena were behind the successful attachment of the NPs onto the MSs. The characteristic macroporosity exhibited by the microspheres is not affected by the loading process, which is essential for an efficient flow of the pollutant solution through the photocatalytic microspheres. A continuous flow type of reactor was employed for the photocatalytic tests, which were consecutively performed first with methyl orange, and followed by paracetamol (20 ppm pollutant aqueous solution), under simulated solar illumination. Apparent rate constants (*k*) normalized to the catalyst TiO_2_ weight, together with the catalyst to pollutant weight ratio, are parameters that enabled us to make a comparative assessment of our technology. The photocatalytic microspheres (TiO_2_ NPs loaded SiO_2_ MSs) developed in the present work, together with the continuous flow set‐up, allowed a similar, or even better photocatalytic activity than other works in the literature (*k* of the order of 10^−2^ min^−1^ g^−1^), but with the advantage of the photocatalyst being easily removed from the reaction medium and more appealing to a real, industrial application.

Inorganic silica meso‐ and macroporous microspheres are a viable and promising platform for photocatalyst immobilization, in the form of robust and efficient photocatalytic systems designed for in‐flow purification of wastewater, through solar‐driven photocatalysis.

## Conflict of Interest

The authors declare no conflict of interest.

## Supporting information

Supporting Information
