# Supplementary material for: Porous Silica Microspheres with Immobilized Titania Nanoparticles for In‐Flow Solar‐Driven Purification of Wastewater
Source: Glob Chall. 2021 Jan 27;5(5):2000116. doi: 10.1002/gch2.202000116 (PMC8101353; doi:10.1002/gch2.202000116)
Supplement: Supplementary file 1 — Supporting Information [file GCH2-5-2000116-s001.pdf]

© 2021 Wiley-VCH GmbH

Global Challenges Open Access

Supporting Information

for *Global Challenges,* DOI: 10.1002/gch2.202000116

Porous Silica Microspheres with Immobilized Titania Nanoparticles for In-Flow Solar-Driven Purification of Wastewater

Ana C. Marques, Mário Vale, Daniel Vicente, Murielle Schreck, Elena Tervoort, and Markus Niederberger*

Porous silica microspheres with immobilized titania nanoparticles for in-flow solar-driven purification of wastewater

Ana C. Marques[1*], Mário Vale[1], Daniel Vicente[1], Murielle Schreck[2], Elena Tervoort[2], Markus Niederberger[2]

[1] CERENA, DEQ, Instituto Superior Técnico, Universidade de Lisboa, Avenida Rovisco Pais, 1049-001 Lisboa, Portugal

[2] Laboratory for Multifunctional Materials, Department of Materials, ETH Zürich. Vladimir-Prelog-Weg 5, 8093 Zürich, Switzerland

SUPPLEMENTARY INFORMATION

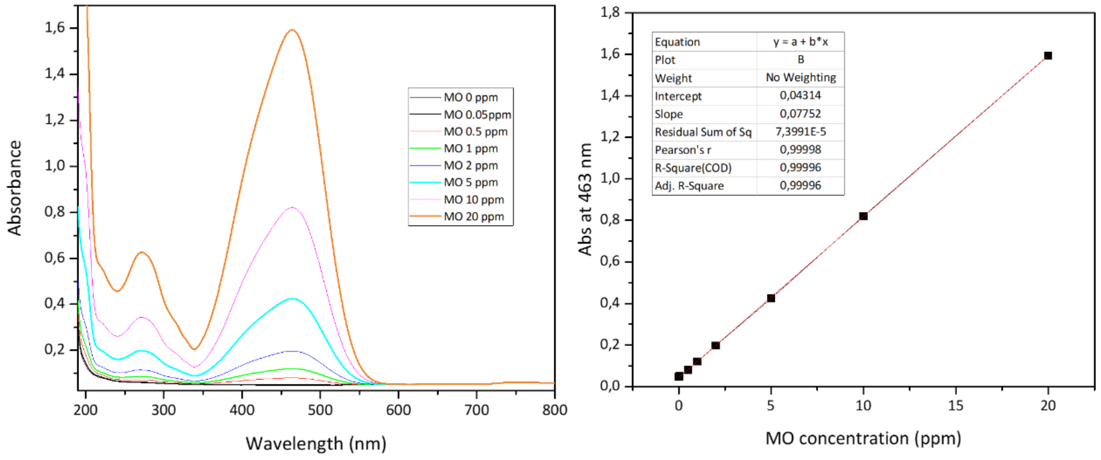

Figure S1. UV-Vis spectra of methyl orange (MO) aqueous solutions at different concentrations indicating two absorption maxima (271 and 463 nm). Calibration curve for MO.

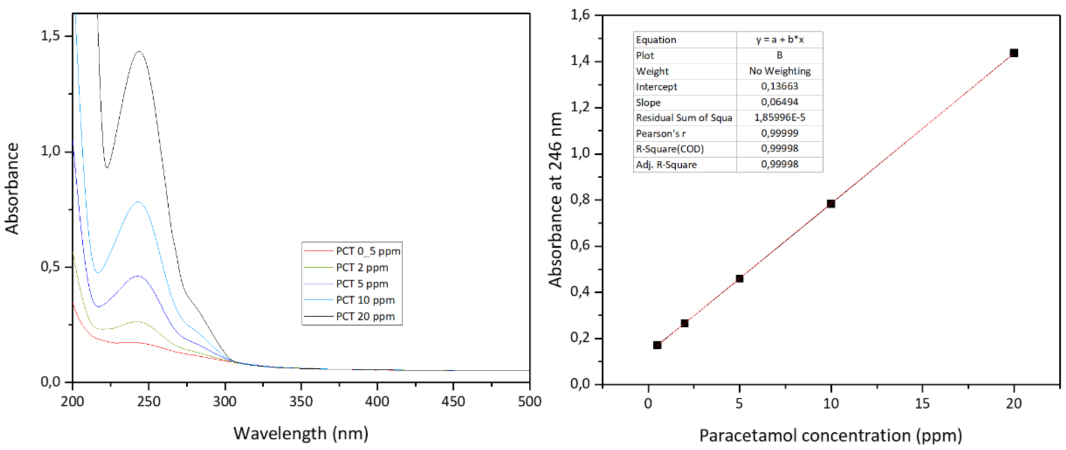

Figure S2. UV-Vis spectra of paracetamol (PCT) aqueous solutions at different concentrations indicating an absorption maximum (246 nm). Calibration curve for paracetamol.

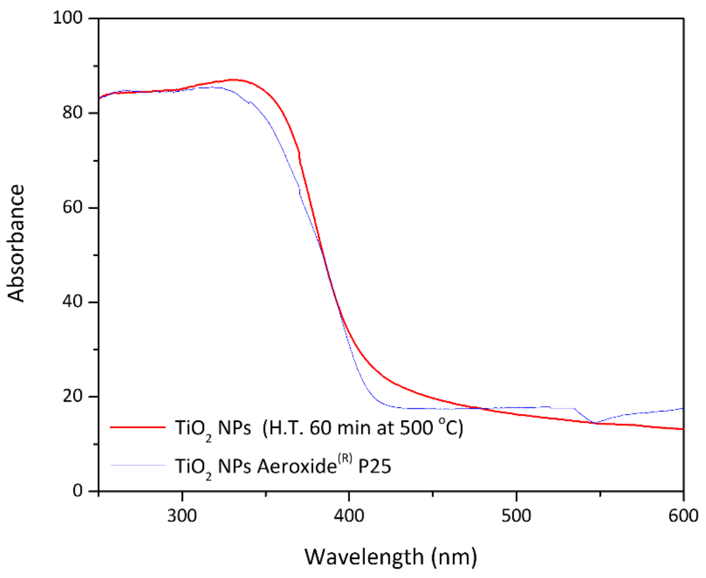

Figure S3. Absorbance spectra for the TiO_2 NPs synthesized in the present work, after heat treatment at 500 ºC for 1h, and for commercially available TiO_2 NPs Aeroxide® P25.

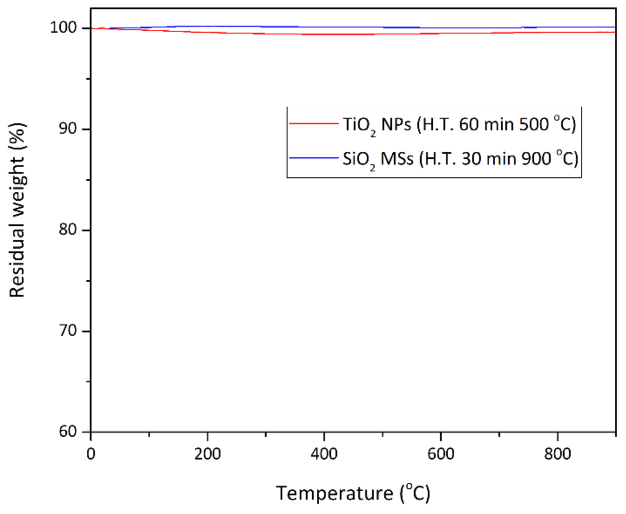

Figure S4. Thermogravimetric curves of the heat treated TiO_2 NPs and SiO_2 MSs, exhibiting their dominant inorganic nature.

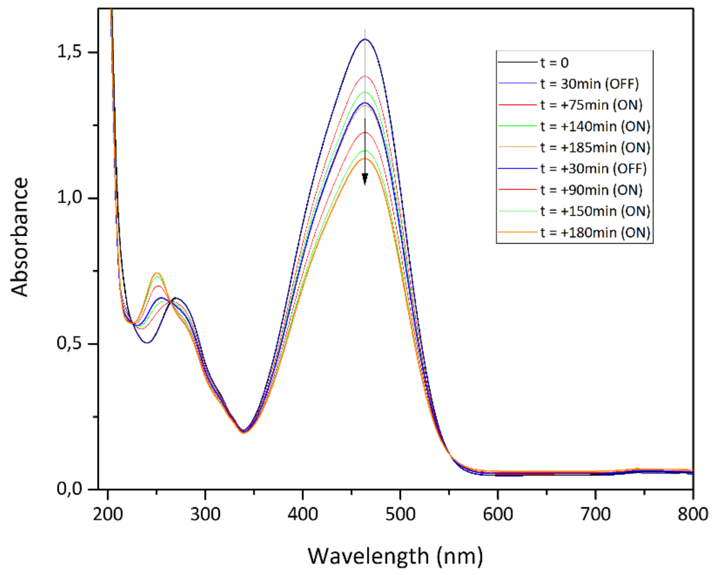

Figure S5. UV-Vis spectra of methyl orange (MO) aqueous solutions at increasing times of solar light exposure. Cycles ON/OFF were done to check for adsorption phenomena. MO degradation does not occur in the dark. Experimental conditions: 25 ºC, pH = 7,

mass$_{(TiO2\ NPs)}$/mass$_{(MO)}$ = 25, 100 mL of 20 ppm MO aq. solution, 300 mg of TiO_2 NPs loaded SiO_2 MSs (66 mg TiO_2), flow = 5 mL/min, irradiance = 1000 W/m^2 (1 sun).

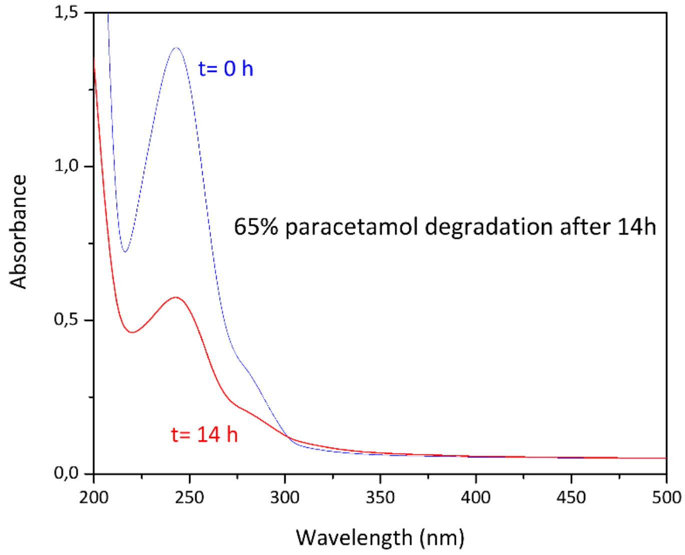

Figure S6. UV-Vis spectra of paracetamol (PCT) aqueous solutions at the beginning and at the end of the photocatalysis test (solar illumination). Experimental conditions: 25 ^{o}C, pH = 7, mass$_{(TiO2\ NPs)}$/mass$_{(PCT)}$ = 26, 100 mL of 20 ppm PCT aq. solution, 300 mg of TiO_2 NPs loaded SiO_2 MSs (66 mg TiO_2), flow = 5 mL/min, irradiance = 1000 W/m^2 (1 sun).
